# Designed for life: biocompatible de novo designed proteins and components

**DOI:** 10.1098/rsif.2018.0472

**Published:** 2018-08-29

**Authors:** Katie J. Grayson, J. L. Ross Anderson

**Affiliations:** 1 School of Biochemistry, University of Bristol, Biomedical Sciences Building, Bristol BS8 1TD, UK; 2 BrisSynBio Synthetic Biology Research Centre, University of Bristol, Life Sciences Building, Tyndall Avenue, Bristol BS8 1TQ, UK

**Keywords:** de novo protein design, synthetic biology, biocompatibility

## Abstract

A principal goal of synthetic biology is the de novo design or redesign of biomolecular components. In addition to revealing fundamentally important information regarding natural biomolecular engineering and biochemistry, functional building blocks will ultimately be provided for applications including the manufacture of valuable products and therapeutics. To fully realize this ambitious goal, the designed components must be biocompatible, working in concert with natural biochemical processes and pathways, while not adversely affecting cellular function. For example, de novo protein design has provided us with a wide repertoire of structures and functions, including those that can be assembled and function *in vivo*. Here we discuss such biocompatible designs, as well as others that have the potential to become biocompatible, including non-protein molecules, and routes to achieving full biological integration.

## 1. Introduction

Designed biomolecular and synthetic components that reproduce or even supersede the functions and activities of natural proteins and enzymes promise to revolutionize synthetic biology, industrial biotechnology and medicine [1,2]. While Nature has undoubtedly provided us with a rich diversity of natural biomolecules, they might not be well adapted for a selected purpose or environment outside of their preferred cellular milieu [3,4]. With artificial components such as de novo proteins, their sequence, structure and biophysical characteristics are selected solely by the designer, conferring, for instance, greater thermal and chemical stability than their natural equivalents and rendering them better suited to our requirements. Chemical activities unknown in Nature may be imposed upon them [5], imparting unique reactivities that may be integrated into the biochemical processes of living organisms. Such endeavours not only result in useful biomolecular or artificial componentry, but also provide powerful, fundamental insights into the engineering of natural biomolecules.

This review focuses on artificial components that are, or have the potential to become, biocompatible. To be defined as biocompatible a component must efficiently perform an intended role *in vivo* and at least be able to interact in a complementary manner with cells or natural biomolecular components. To satisfy a more stringent definition of biocompatibility, the components—or the biosynthetic pathway that produces them—should be genetically encoded or imported directly into the cell, and they should be fully assembled and functional *in vivo,* without any significant deleterious effects. For cofactor-dependent proteins and enzymes, this inevitably requires post-translational insertion of small molecules such as hemes and flavins to impart the desired functionality.

With such biocompatible components, there is then an opportunity to design systems where natural and synthetic components work synergistically to expand the range of possibilities offered by entirely natural or entirely synthetic systems [6]. Synthetic molecules that can be produced by living organisms also present the possibility of ‘eco-friendly’ manufacturing, negating the need for expensive synthetic processes [4].

Translating a particular function from a natural protein to a synthetic element is a challenge, and achieving biocompatibility is a further hurdle due to the immense complexity, diversity and specificity of cellular processes [7]. Currently, the components that most fulfil these requirements are de novo designed proteins, although there are other chemical entities that, with further development, could become biocompatible. Here we will discuss recent developments in the design of de novo proteins and non-natural elements that reproduce natural biomolecular functions, with a particular focus on biocompatibility. This review is not intended to be exhaustive, but key examples have been selected to illustrate the topics covered. We will also look to the future and highlight research that lays the groundwork towards the use of synthetic elements *in vivo*.

## 2. Protein scaffold design

Before function can be conferred onto an artificial protein, robust yet mutatable protein scaffolds must be designed. These proteins may directly mimic existing, natural structures, or adopt completely new folds. Simplicity and tolerance to mutations are key to designing a protein scaffold; a protein that is highly tolerant to mutation while largely retaining its tertiary structure allows the designer to alter or improve function in a tractable and facile manner. A well-defined structure and amenability to characterization techniques are obviously ideal in such a process, though the ability to design and predict de novo protein structures with atomic detail remains a significant challenge [1]. It is also not always easy to predict how changes to a protein's amino acid sequence might affect its stability, structure and function, therefore simplicity is key. For this reason, the redesign of natural proteins may not be the simplest approach to achieving new function, although this has been a fruitful area of research.

The complex network of interactions found in natural proteins has arisen through millennia of natural selection. These networks arise as amino acids within the protein become irreversibly co-dependent, resulting in a Muller's ratchet-like accumulation of fragility and resistance to modification and change [8,9]. It is usually challenging to wholesale identify the functional roles of any one amino acid, or to discern which specific amino acids support a given function. Evolutionarily naive, de novo designed proteins can eliminate this problem and provide a simple framework on which to build function [10,11]. Ultimately, a protein is designed and constructed in which the roles of each individual amino acid are more easily determined and controlled, and a more tractable design process can thus be implemented.

Given the relative simplicity of designing helical peptides and small helical bundle proteins, there are now many examples of functional de novo designed proteins whose scaffolds are constructed from alpha helices [11–15]. The design principles for assembling helices are elementary: two turns of an alpha helix can be formed by a heptad of amino acids with helical-forming propensities (e.g. alanine, leucine, glutamic acid), and repeating heptads of such residues will extend the helix length as required [16]. To form larger oligomeric helical assemblies such as unlinked coiled coils, a defined hydrophobic/hydrophilic periodicity is imprinted on the heptads, dictating the size and orientation of the hydrophobic face, which in turn defines the oligomeric state of the assembled peptides [17]. These coiled coils are often used in de novo protein design and consist of bundles of two or more helices that form a rope-like superhelical structure with well-defined, interhelical knobs-into-holes packing [18–20]. The folding of coiled coils, and soluble proteins in general, is driven by the favourable entropy change when water is expelled from the interior of the folding protein [16,21]. Simple design principles similar to those employed in coiled-coil heptad repeat patterns can also be used to construct elementary 4-helix bundles that form discrete and stable scaffolds that do not necessarily conform to coiled-coil structural parameters [16,22]. Most published de novo coiled coils are parallel with respect to each other, and because their N-termini are co-located, it is not possible to genetically loop them together with short peptide sequences for expression as a single-chain protein. However, in designs with helices that assemble in an antiparallel manner, the helices can be linked through simple loops containing residues with low helical-forming propensities, such as glycine and serine [22,23]. Therefore, in these cases, a single-chain helical bundle can be genetically encoded that not only facilitates *in vivo* protein expression, but also allows the cross-bundle sequence symmetry to be broken [23,24].

**Figure 1.**
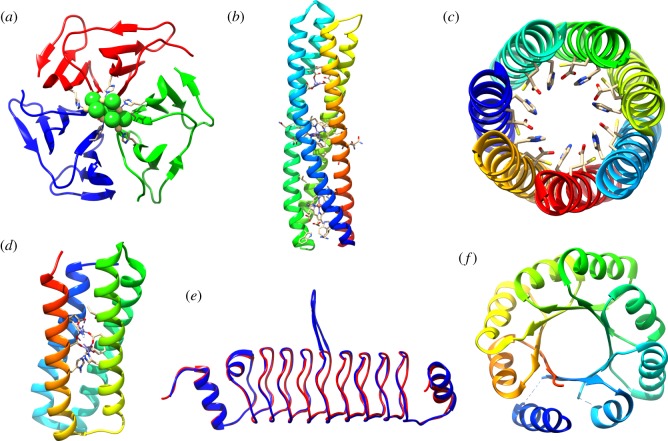
The diversity of de novo designed protein structures. (*a*) Pizza variant, nvPizza2-S16H58, which coordinates a CdCl_2_ nanocrystal [26]. PDB: 5CHB. (*b*) De novo designed reaction centre with heme B, synthetic Zn porphyrin and Zn(II) cations [27]. PDB: 5VJS. (*c*) Catalytic helical barrel, CC-Hept-I18C-L22H-I25E. Catalytic triad residues are shown [28]. PDB: 5EZC. (*d*) DFsc-Zn(II)_2_ used by Ulas *et al*. [29] for semiquinone radical stabilization. PDB: 2LFD. (*e*) Designed beta solenoid proteins, SynRFR24.1 (red, PDB: 4YC5) and SynRFR24.t1428 (blue, PDB: 5DNS) [30]. (*f*) sTIM-11 [31]. PDB: 5BVL.

Even within a simple α-helix bundle, protein backbones can have highly variable geometry in which each amino acid can adopt many different side chain conformations. To remedy this, recent research by the Baker group focused on the design of protein interfaces with regular networks of hydrogen bonds that specifically interact in a modular way, similar to the base-pairing of DNA [25]. The simplicity of α-helix bundle proteins is in many ways an advantage over more complex structures. However, the design of larger structures, including those that involve β-sheets, may allow us to access a wide range of functional capabilities. Existing de novo protein designs form a diverse range of structures, some of which are shown in figure 1.

The use of repeat sequences in protein design brings the advantage of modularity and allows the construction of larger, more complex scaffolds. Repeat proteins are prevalent in Nature, and present attractive targets for protein design [32]. For example, repeat five-residue (RFR) beta-solenoids can tolerate substantial variations including mutations to the loop regions that link together the individual beta-helix subunits [30]. To capitalize on the potential of these versatile scaffolds, MacDonald *et al.* have developed computational methods which were used to calculate de novo backbones without using existing sequences of natural proteins [33–35]. The authors then created a set of genetically encodable, de novo RFR-fold proteins with variable loops, and even whole protein insertions in the loop regions [30] (figure 1*e*).

The TIM-barrel fold is possibly the most prevalent protein topology found in natural enzymes, comprising eight α-helices surrounding eight β-strands in a closed toroid [36]. Despite the structural similarity of TIM-barrel enzymes, there is very little sequence conservation across the many superfamilies that adopt this topology [31,37,38]. While the TIM-barrel structure appears complex, there is much interest in the design of this topology de novo, owing to the functional diversity that might be tangibly available to the designer. With recent advances in computational protein design, the Baker group has created a series of genetically encodable TIM-barrel variants. One variant, sTim-11 (figure 1*f*), was crystallized to reveal excellent structural fidelity to the original design. For simplicity, the group aimed to design a structure with fourfold symmetry, the maximum possible in this design. sTim-11 features four repeating motifs forming a structure which is thermostable and reversibly folds after denaturation by guanidinium chloride and temperature, providing a unique structure for the precise placement of catalytic amino acids [31].

While the vast majority of designed proteins are soluble, natural membrane proteins have an array of functions that are worth replicating in de novo designed proteins, including receptors, transport in and out of the cell, and roles in photosynthesis. However, the design of de novo membrane protein scaffolds is hindered by the relatively small proportion of solved membrane protein structures compared with soluble proteins. In contrast to soluble proteins, designing a membrane protein that assembles, localizes and functions as intended is significantly more challenging. While the basic design principles for de novo designed membrane proteins are well established [39], in practice it is often the case that the protein is incorporated into inclusion bodies [40] (which is not ideal for *in vivo* function), or that their low yields [41] and poor solubility can complicate downstream study.

Despite these difficulties, there have been significant advances in de novo membrane protein design in recent years, and achieving full, functional, biocompatibility is in sight. Many de novo membrane protein designs are made via peptide synthesis (see §4.5 De novo designed membrane pores) [13], although amphiphilic maquettes can be expressed in *Escherichia coli* and human embryonic kidney cells (see §4.2 Light-responsive artificial proteins) [40]. Recent research by the Baker group has led to the design of de novo multipass membrane proteins that locate to the membrane of *E. coli* and human kidney cells, with crystal structures revealing fidelity to the intended design [42].

For a review of de novo designed protein structures see Huang *et al.* [1]. Polymeric de novo peptides, such as the catalytic beta amyloids designed by the Korendovych group, are probably incompatible with the cell and therefore beyond the remit of this review; for a review on this topic and other catalytic peptide assemblies, see [43].

Function can be incorporated into a de novo protein design through the use of cofactors; however, designing a highly specific cofactor-binding site is not always straightforward. Amino acid side chains can directly coordinate metal ions [44], but when the metal ion is part of a larger structure, such as heme, or in the case of other bulky molecules such as flavin, the situation becomes more complex. While basic design principles have been uncovered, progress in this area has been slow. Research by the Koder and Noy groups involved the scanning of databases of natural proteins to identify consensus sequences and geometric properties for heme and chlorophyll-binding sites using histidine residues [45,46]. While there are computational methods in place for the design of cofactor-binding sites (for metal-binding sites, see [44]), further progress is required. Furthermore, when trying to replicate the function of, for example, light-harvesting proteins which bind multiple interacting cofactors, the situation becomes more complicated still. Not only must the cofactors be specifically bound, in the correct orientations with the correct properties, but also their interactions and properties must be tuned.

## 3. Fully biocompatible de novo designed proteins

### 3.1. Life-sustaining functions

**Figure 2.**
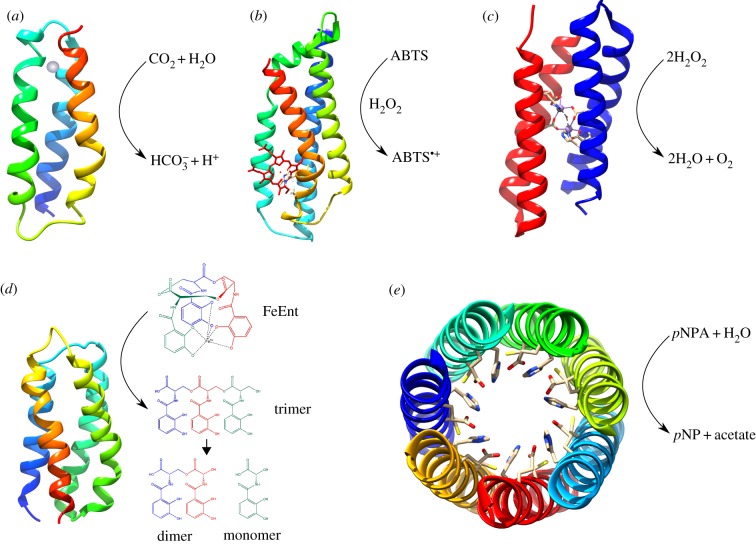
A selection of catalytic de novo proteins. (*a*) Representation of the structure of a de novo protein which performs carbonic anhydrase activity. The solution nuclear magnetic resonance structure of the α3D scaffold (PDB: 2A3D [53]), was modified to bind zinc (grey) and hydrate CO_2_ [54]. (*b*) Molecular dynamics simulation model of C45, which can catalyse the oxidation of a range of small molecules, including 2,2′-azino-bis(3-ethylbenzothiazoline-6-sulfonic acid) (ABTS) [12]. (*c*) The de novo protein Dft2 was modified to bind manganese and perform catalase activity [55,56]. The crystal structure shown is that of the variant, P0 (PDB: 5C39); variants with two and three manganese-binding sites exhibit higher activity. (*d*) A 4-helix bundle library protein, Syn-F4, which performs enantioselective hydrolysis of ferric enterobactin (FeEnt) [57]. As no structure is available of this protein, the structure shown is a representative 4-helix bindle from the Hecht lab (PDB: 2JUA). (*e*) Crystal structure of the heptameric coiled-coil CC-Hept-I18C-L22H-I25E with hydrolase activity towards *p*-nitrophenyl acetate (*p*NPA). Catalytic triad residues are shown. (PDB: 5EZC) [28].

The Hecht group has explored whether proteins with life-sustaining properties can spontaneously arise from combinatorial libraries of de novo designed proteins. These libraries were created using a simple binary code strategy, where amino acids are considered as either polar or non-polar in a repeating pattern, ensuring the resulting proteins were folded into stable, discrete 3D structures [22,47]. The libraries were screened for function through expression in various *E. coli* auxotroph strains. Despite the stochastic nature of their sequence selection, several de novo proteins were capable of rescuing specific knockout strains [48]. Some of these de novo sequences have been demonstrated to act on gene regulation [49], in one case allowing the cells to grow on toxic levels of copper [50]. One particular de novo protein, SynGltA, could rescue a citrate synthase gene deletion mutant unable to catalyse the first reaction of the tricarboxylic acid cycle. It was found that SynGltA does not reproduce the catalytic activity of citrate synthase, but instead upregulates a pathway which includes the promiscuous enzyme methylcitrate synthase, producing sufficient citrate to rescue growth [51]. This highlights the potential roles de novo proteins might have in ‘rewiring’ gene pathways and metabolism in auxotrophs [51]. In addition, a de novo catalytic protein, Syn-F4, has been developed from the library protein, Syn-IF. Syn-IF rescued two different *E. coli* auxotroph strains, but did not appear to have a catalytic role [52]. Following random mutagenesis and selection of protein variants that could more rapidly rescue the auxotroph strain, the variant Syn-F4 was found to have catalytic activity *in vivo*, namely the enantioselective hydrolysis of ferric enterobactin (figure 2*d*) [57].

### 3.2. Therapeutic functions

**Figure 3.**
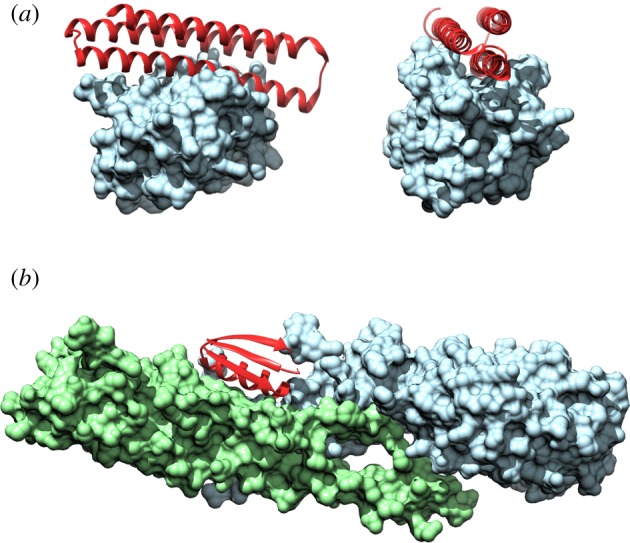
Crystal structures of de novo inhibitors binding to their targets. Crystal structures of inhibitor complexes. (*a*) Inhibitor peptide αMCL1 (red) binds the human BCL2 homologue, Mcl-1 (blue), with picomolar affinity [60]. PDB: 5JSB. (*b*) Inhibitor peptide HB1.6928.2.3 (red), which can bind influenza haemagglutinin [59]. PDB: 5VLI.

Man-made biocompatible entities offer opportunities for designing therapeutic and diagnostic agents to combat disease, an avenue the Baker group has explored [58–61]. Mimics of pro-apoptotic proteins have been used as treatments against diseases in which apoptosis is dysregulated, such as cancer. One such approach is to mimic proteins that can inhibit, through binding, BCL2 family pro-survival proteins that are expressed in many cancers [60]. Members of this family have very high sequence homology and structures, so specific BCL2 binding is a challenge [60]. The Baker group has created de novo proteins which bind to the BH3 binding groove of certain pro-survival proteins. Initially, a 3-helix bundle protein, BINDI, was designed as an inhibitor of BHRF1, an Epstein–Barr BCL2 homologue [61]. This scaffold was subsequently modified to produce variants that could each bind one of the six human pro-survival BCL2 family proteins (figure 3*a*), and can be expressed in human cancer cell lines [60]. Rosetta Monte Carlo sequence design was used to design the proteins, which have three helices, one with a central BH3 motif. The two remaining helices were designed to aid specificity and stability. The de novo protein scaffold contacts regions of the BCL2 proteins that differ in sequence between family members. Following computational design, two of the designed proteins had high specificity and affinity for their targets, the remaining four provided good starting targets for *in vitro* optimization. Thus, these de novo proteins form a set of molecular probes which can be used for various purposes, including determining which of the BCL2 proteins are involved in individual cancers, and aiding understanding of the mechanisms of mitochondrial apoptotic pathways [60].

The development of increasingly higher-throughput and computational methods has been a great aid to the field of de novo protein design, particularly when it comes to designing therapeutics [62]. In a recent study, the Baker group has developed a high-throughput computational method using Rosetta to design small protein binders of specific therapeutic targets, in this case influenza A H1 haemagglutinin (figure 3*b*) and botulinum neurotoxin B [59]. This method allowed the simultaneous study of thousands of small protein designs, in which target binding and protein folding were characterized. Of the 22 600 starting proteins, more than 10% exhibited high-affinity binding to their targets; one protein was effective against influenza infection in mice, even when administered post-exposure. The proteins function in a similar way to neutralizing antibodies, which bind to and neutralize the activity of the target infectious agent or antigen, often preventing its entrance into the cell. The designs are highly specific, stable (including thermostable) and do not provoke an immune response. Although these approximately 40 residue proteins are genetically encodable, they can also be chemically synthesized, presenting the opportunity of chemical modification to expand their function. This high-throughput method is a promising approach to the production of new therapeutics and diagnostic tools for a wide range of targets [59].

There is much interest in the development of protein cages, both natural and artificial, for the delivery of therapeutic molecules [63], for example by mimicking the function of a virus. Synthetic nucleocapsids designed by the Baker group can be fully assembled with their mRNA genome in *E. coli* cells and, following injection, persist in the circulation of mice for several hours [64]. Computationally designed combinatorial libraries were produced and subjected to rounds of directed evolution to select for certain properties common to natural viruses: a well-packaged genome, resistance to nucleases and persistence in *in vivo* blood circulation. These nucleocapsids provide a tailorable platform for future applications such as therapeutic purposes, while minimizing the complexity found in naturally evolved viruses.

### 3.3. Interaction of de novo proteins with cellular machineries *in vivo*

To be fully biocompatible, a de novo protein must not only be expressed *in vivo*, but also interact productively with natural cellular machineries, while undesirable interactions are minimized. The functionalities of natural systems may be harnessed, for example, by transporting man-designed elements to the desired location within the cell. In recent research the *E. coli* twin-arginine translocation (TAT) apparatus, whose quality control mechanism will only allow the export of fully folded proteins across the cytoplasmic membrane, could ‘read’ the folding state of a completely artificial heme-binding protein and translocate it to the periplasm [65]. While the bacterial Sec system has been proved capable of transporting de novo proteins in an unfolded state [24], the TAT system may be able to transport other de novo proteins that must fold in the cytoplasm prior to translocation. Beyond transport, there are other cellular processes that may be exploited for the modification of de novo designed proteins, such as the rich diversity of natural post-translational modifications.

Cells can selectively insert cofactors, for example, using accessory proteins and enzymes in what can be quite complex, multistep pathways. Amino acids from the polypeptide backbone may be modified to produce *in situ* cofactors, such as the covalently attached quinones (e.g. the topaquinone cofactor found in natural copper amine oxidases [66]), which are often formed through the modification of tyrosine or tryptophan. Other modifications include catalytic activation or suppression through phosphorylation, and glycosylation, in which carbohydrate is covalently attached to the protein to aid stability and modulate activity.

In recent research, the Anderson group has designed and characterized an artificial heme C-containing oxidoreductase, C45, which makes use of the native *E. coli* cytochrome *c* maturation (Ccm) machinery to assemble the protein in its active form *in vivo* [12]. The natural oxidoreductases are a functionally diverse superfamily of enzymes, performing a plethora of chemical transformations, and there has been much interest in replicating and exploiting their functions through de novo protein design [14,67,68]. Owing to their catalytic power and potential utility in chemical synthesis and biotechnology, there has been particular interest in the oxygenases and peroxidases, catalysing the controlled insertion of oxygen into carbon–hydrogen bonds and the coupled oxidation of small molecules/reduction of hydrogen peroxide, respectively. Within these enzyme classes, many contain catalytically versatile heme cofactors that, despite the wide range of chemistries displayed by heme-containing enzymes, use essentially the same reactive intermediates to facilitate the diverse array of chemical transformations [69]. While artificial peroxidases have been developed [70,71], heme-containing oxygenases have proved more difficult. Heme can be spontaneously and non-covalently incorporated into proteins that are expressed *in vivo* [72] and it is possible to build a simple heme-binding site within a de novo protein scaffold using histidine residues to coordinate the heme iron [67]. However, covalently appending heme to the protein backbone ensures secure and practically irreversible attachment of the cofactor, and can facilitate the design of more sophisticated de novo assemblies [24]. C45 has arisen from the iterative improvement of oxygen-binding *c*-type cytochrome maquettes which contain a CXXCH motif on the protein backbone for heme C insertion by the cytochrome *c* maturation (Ccm) apparatus [12,24,68]. Unlike these catalytically inactive maquettes with bis-histidine heme ligation, C45 contains a monohistidine ligated heme, allowing molecules such as hydrogen peroxide to bind in the vacant heme coordinate site and become activated towards simple substrate oxidation reactions. C45 is catalytically promiscuous and can oxidize a range of small molecules (figure 2*b*) [12]. With regard to catalytic efficiency, C45 matches the activity of natural peroxidases against certain substrates. As C45 is produced in its functional form in *E. coli*, it can probably perform this type of peroxidase catalysis *in vivo*.

## 4. De novo designed proteins: *in vitro* assembly and function

Many de novo proteins can be genetically encoded as single-chain proteins that can be expressed by living cells [23,24]. However, the designs may often incorporate functional elements that are not synthesized or constructed by the host cell and must currently be added *in vitro* [23]. Therefore, many genetically encodable artificial proteins are expressed in *E. coli* and subsequently purified for further assembly, study and analysis [73,74]. Future challenges include the creation of new biosynthetic pathways to synthesize novel cofactors in cells, and the design of specific binding sites with high affinity for the intended cofactor. To date, de novo proteins have been designed to mimic a variety of natural protein functions; advances in the last few years are discussed below, with perspectives on biocompatibility.

### 4.1. Artificial enzymes

In Nature, protein catalytic function is often complex, with many factors working in concert to allow efficient chemistry and to ensure the reaction is thermodynamically favourable. These factors may include diffusion of the substrate and product in and out of the active site, quantum tunnelling effects, transition state stabilization, specificity and concerted protein dynamics [75–79]. In many cases, the precise alignment and proximity of a substrate molecule and active-site amino acid side chains is important [80,81]. Imparting of catalytic function onto a de novo scaffold can therefore be a challenge. There are many examples of catalytic de novo proteins and peptides, and these are reviewed in Zozulia *et al.* [43], some of which are shown in figure 2. A successful route to achieving catalysis has included the use of metal cofactors to perform chemistry, for example the aforementioned heme C-containing maquette, C45, which performs efficient catalysis without a highly specific substrate binding site [12,82] (figure 2*b*). However, in many cases it has been necessary to incorporate the cofactors *in vitro,* and further research and development is required to enable full functional assembly *in vivo*.

As most natural diiron-containing enzymes contain at their core a 4-helix bundle that binds the two iron ions necessary for their diverse and powerful enzymatic functions, these enzymes were early targets of de novo protein design [83]. The natural enzymes catalyse a variety of substrate oxidations and oxygenations, including the thermodynamically challenging hydroxylation of methane [84]. Within the iron-binding core of the natural enzymes, the iron ions are generally coordinated by two histidine and four carboxylate residues [85]. The DeGrado group has performed much work on its due ferri (DF) de novo proteins, whose simplicity has provided significant insight into the natural non-heme diiron enzymes; for a review of artificial diiron-oxo proteins of the DF family, see Chino *et al*. [85]. Iterative design processes on the single-chain protein, DFsc, have produced variants with increased solvent and substrate accessibility, and modified reactivity—from hydroquinone oxidation to selective *N*-hydroxylation of arylamines [86]. The incorporation of a third ligating histidine residue to the variant G4DFsc altered the enzyme's function, mimicking the active site of *p*-aminobenzoate *N*-oxygenase, a natural diiron protein [86]. The modified protein gains oxygenase activity towards *p*-anisidine, while 4-aminophenol oxidation is disfavoured [86]. As the coordination of one iron site is saturated, a slower substrate binding rate is observed, inhibiting oxidase reactivity [87,88]. While both G4DFsc and 3His-G4DFsc bind *p*-anisidine near the biferrous site, the geometry of this site is differentially perturbed in such a way as to influence reactivity. While these proteins are expressed in *E. coli*, they are reconstituted with iron *in vitro*, and would probably require a significant increase in iron affinity to promote *in vivo* assembly and activity.

Other metal ions have been effectively used as catalytic cofactors in de novo designed proteins. The Pecoraro group has designed a carbonic anhydrase (CA) mimic, with a zinc-containing active site analogous to that of CA II, but in a 3-stranded α-helical coiled coil as opposed to the β-sheets found in CA II. While the efficiency of CO_2_ hydration is within two orders of magnitude of CA II, it is a parallel assembly whose symmetry hinders improvements and is not manufacturable *in vivo* [89]. In a more recent study, the Pecoraro group modified an existing single-chain DeGrado scaffold, α3D [53,90], to bind zinc and hydrate CO_2_ (figure 2*a*) [54]. Although the catalytic efficiency is 1400-fold less than that of CA II and 11-fold less than that of CA III, the simple scaffold and iterative design strategy will facilitate the design of improved function. The protein can be expressed in *E. coli*, laying the groundwork for future design of *in vivo* activity [54]. α3D has been modified to bind various other metal ions to perform functions other than catalysis, such as copper [91] (see §4.3 Designed electron transport proteins) and heavy metals [92] (see §4.4 Artificial metal-sequestering proteins).

Many natural proteins contain manganese cofactors, which have a wide variety of functions, notably the evolution of molecular oxygen. Allen and colleagues have altered a DeGrado DF protein, Df2t, to produce a series of four helix bundle proteins, made up of two monomers, with dinuclear manganese (Mn) binding sites, analogous to that found in Mn-catalase (figure 2*c*) [55,56]. These proteins exhibit catalase activity, converting hydrogen peroxide into molecular oxygen. Variants, ‘P1’ and ‘P3’, with three metal-binding sites exhibited higher activity than those with fewer binding sites, ‘P0’ (one site) and ‘P2’ (two sites). The apo-proteins were purified from *E. coli*, and the Mn cofactors were incorporated *in vitro* through incubation with MnCl_2_. These proteins can also transfer electrons to bacterial reaction centres (RCs), discussed in §4.2 Light-responsive artificial proteins.

Catalytic function has also been integrated into de novo designed scaffolds without the use of cofactors. Burton *et al.* [28] reported a de novo helical barrel with active sites that mimic those of natural cysteine/serine hydrolases, in which amino acid side chains alone catalyse the reaction (figure 2*e*). This structure comprises seven helices forming an 8 Å diameter channel, with each helix featuring a cysteine–histidine–glutamic acid catalytic triad in the barrel lumen, resulting in a total of seven catalytic triads in the fully assembled protein. A combination of rational design and iterative strategies was used in which the design was fully characterized with each additional mutation. Although there have been other de novo protein hydrolase designs containing Zn^2+^ with higher catalytic efficiency [93,94], this protein forms a unique structure with a highly mutatable de novo scaffold, and sequential rounds of directed evolution may aid further enhancement of its catalytic ability. While this protein was produced using peptide synthesis and is a parallel assembly and not a single-chain construct and therefore cannot be constructed *in vivo*, it is a good recent example of how catalysis may be performed in a de novo designed protein without the requirement for cofactors.

#### 4.1.1. Steps towards catalysis in de novo designed proteins

With the exception of a few notable examples, most de novo enzymes fall short of the catalytic efficiencies exhibited by natural enzymes. A richer understanding of how natural enzymes work and how to import these functions into de novo designed elements may enable us to perform more complex or tuneable reactions. This section briefly discusses some current ways in which de novo design is being used to understand some features of natural enzyme catalysis with the hope that we may be able to use this new knowledge to create improved artificial protein catalysts.

Enzymes can stabilize high-energy intermediates, and some make use of radicals, which must be controlled to avoid damage to the enzyme. It is not well understood how natural proteins can stabilize these unstable species, and Tommos and colleagues have used de novo proteins as models to study the stabilization of amino acid and mercaptophenol radicals [95–97]. The DeGrado group has used rational protein design to stabilize ortho-semiquinones, common radical intermediates found in natural catalysis and redox processes [29]. In this study, a 4-helix bundle protein, DFsc, was used to bind Zn(II), to which the semiquinone was stabilized through binding (figure 1*d*). The location of the Zn(II) in the hydrophobic cleft of the protein excludes the bound semiquinone from the bulk water. At room temperature and in aqueous medium, binding of the semiquinone radical, SQ•, was favoured over binding of the more stable QH_2_ and Q forms. The design principles used here, in which the radical is stabilized through metal–ligand interactions and burial of hydrophobic groups, may allow us to design artificial enzymes that can perform more complex chemistry [29].

The rate of an enzymatic reaction in a de novo designed active site is often limited by imperfect geometry. Therefore, the ability to customize a ligand-binding pocket towards high specificity is an advantage. The Baker group investigated using beta sheets to custom-design backbones for binding a specific ligand [98]. Furthermore, in natural proteins, amino acids far (10–20 Å) from the active site can contribute to ligand-binding geometry, and DeGrado and colleagues [99] have designed a de novo protein, PS1, with this in mind. PS1 was designed to mimic natural proteins with apolar folded cores which support cofactor-binding regions. Prior to cofactor binding, the binding region is flexible relative to the tightly packed core. On binding the cofactor, the entire protein is tightly packed and stabilized. PS1 is faithful to the design to sub-Å level, and binds a non-natural porphyrin with high thermostability. The success of this approach is promising for the design of improved catalytic de novo proteins, and their structural characterization [99].

### 4.2. Light-responsive artificial proteins

Natural photosynthesis is a highly organized process using pigment–protein complexes to harvest light energy, which is ultimately used to power ATP synthesis. There are many components in natural photosynthetic pathways working synergistically to ensure efficiency and productivity while maintaining the ability to quickly adapt to changing environmental conditions. Although complex, photosynthesis provides a rich source of natural engineering principles from which to draw inspiration for the design of functional de novo proteins. Ultimately, the de novo design of photosynthetic proteins may allow the construction of customizable, modular photosynthetic pathways that are adaptable to and stable within the desired conditions, to light-power the production of valuable products.

Many of the key proteins involved in photosynthesis are membrane proteins. Therefore, efforts have been made to design artificial transmembrane proteins that can emulate the functions of their natural counterparts. Artificial membrane proteins can bind a variety of cofactors such as heme, Zn- and Ni-bacteriochlorophylls, and synthetic tetrapyrroles [100–102]. Amphiphilic maquettes consist of two distinct continuous hydrophilic and lipophilic domains, the latter being of a suitable length to span a lipid bilayer [100,101]. Until recently, amphiphilic maquettes were constructed from self-associating, unlinked helical peptides; however, to break symmetry and therefore increase mutability, a single-chain amphiphilic maquette has been designed [40]. This maquette is expressed in *E. coli*, and*, in vitro*, can bind multiple *b*-type hemes and photoactive Zn protoporphyrin IXs, potentially capable of supporting a light-activatable intra-protein electron transfer (ET) chain. Although this protein forms inclusion bodies when expressed in *E. coli*, the authors ultimately aim to express and assemble these proteins *in vivo* with natural cofactors [40].

Despite advances in designing de novo membrane proteins, it remains simpler to design and work with soluble proteins. Membrane proteins require solubilization with detergents, particular design principles for folding and membrane insertion [103] and often more complex purification protocols than soluble proteins. There is therefore increasing interest in creating water-soluble proteins that perform the function of proteins found in natural photosynthesis, recent examples of which are detailed below.

Photosynthetic organisms tend to occupy particular spectral niches depending on the absorbance of their biosynthesized pigments, and therefore do not exploit the full range of the available solar spectrum [104]. It would thus be exceptionally beneficial to design artificial light-harvesting pathways that could use a greater range of solar energy [105], and there has been a corresponding interest in the design of artificial proteins that selectively bind natural or artificial light-harvesting molecules. Maquettes have been designed to bind light-active chlorins [106,107]: these de novo proteins afford the protein engineer a greater freedom for design than natural light-harvesting proteins, and may be customized to absorb specific wavelengths of light. The hydrophobic nature of many photoactive tetrapyrroles, both natural and synthetic, can render binding to artificial proteins problematic. Natural proteins often use accessory proteins to obfuscate this problem, but a less complex approach would be beneficial to the assembly of de novo proteins. Successful methods in incorporating such hydrophobic molecules into soluble proteins have included the use of detergents [108] or water-in-oil emulsions [109], although other strategies would need to be developed to achieve this *in vivo*. One approach used in previous work regarding the incorporation of chlorophyll (Chl) and bacteriochlorophyll (BChl) into soluble de novo proteins involved the removal of their hydrophobic tails to improve solubility and prevent aggregation [106,107,110]. However, to best make use of the wide range of synthetic and natural light-harvesting pigments (e.g. synthetic chorins and bacteriochlorins) available for use in synthetic systems and whose solubility is variable, we must understand how to effectively incorporate them into the desired protein scaffold without the need to modify the properties of the molecule. Kodali *et al.* [111] have recently demonstrated how to strike a balance between the hydrophobic and hydrophilic nature of the cofactors by producing a soluble light-harvesting maquette which partitions the non-polar region of amphiphilic tetrapyrroles into the interior of the 4-helix bundle, while the polar portion is exposed to the aqueous environment. In this work, the authors used Zn tetraphenyl porphyrins and Zn chlorins whose solubility in different environments was altered through substitutions at the meso-position of the tetrapyrrole ring. These porphyrins were then titrated into a maquette with ligating histidines to determine the optimum cofactor characteristics for binding. Furthermore, within the same maquette, it is possible to include ligating histidines with different binding affinities to incorporate more than one cofactor type into the same scaffold [111].

To achieve biocompatibility, artificial components must work in symbiosis with natural proteins, and functional interactions between man-made and natural proteins may allow us to access functions beyond the current capabilities of de novo protein design alone. One approach has been to create natural–artificial protein chimeras. The Noy group fused a domain of a natural phycobiliprotein with a de novo 4-helix bundle binding a light-active zinc porphyrin or bacteriochlorin [112]. The direction of Förster resonance energy transfer (FRET) between the fusion domains could be altered depending on the particular pigments bound. Such directionality is highly important in natural light-harvesting proteins, ensuring light energy is captured efficiently. In recent research, Mancini *et al.* [113] have created a light-harvesting/energy transfer fusion between a natural bilin-binding protein, CpcA and a 4-helix maquette featuring two photoactive tetrapyrrole-binding sites. The first cofactor-binding site of the maquette can ligate Zn-tetrapyrroles through a histidine residue in the hydrophobic interior of the maquette. The hydrophobic regions of the Zn-chlorin are buried, while maintaining close proximity to the phycobiliprotein for efficient FRET. The second binding site within the maquette is a cysteine residue which facilitates covalent attachment of a synthetic maleimide-functionalized bacteriochlorin, which buries itself in the maquette interior. The entire construct, fully assembled with cofactors, covers a large portion of the UV and visible absorbance spectrum. It can capture light energy and perform multistep excitation energy transfer from the natural bilin-binding protein to the tetrapyrroles of the synthetic protein. These studies demonstrate how natural and synthetic proteins may ‘work together’ for multistep excitation energy transfer, and present tailorable light-harvesting properties due to the option to incorporate different cofactors that absorb different wavelengths of light. Building on this work, de novo proteins with more complex systems of light-active cofactors may be created to more closely mimic natural light-harvesting proteins which can possess a higher number of bound pigments per polypeptide chain.

Though these artificial proteins can be genetically encoded, the functional photoactive complexes are currently assembled *in vitro*. To achieve full biocompatibility, these proteins must bind their photoactive prosthetic group *in vivo*; this can be achieved through the specific binding of photoactive molecules either endogenous to the cell, supplemented in culture or produced from a recombinantly expressed biosynthetic pathway *in vivo*. Mancini *et al.* [113] report that chlorins native to the photosynthetic cyanobacterium *Synechocystis* sp. PCC603 bind maquettes expressed *in vivo*. Phycobilins can also be attached onto maquette cysteine residues in *E. coli* when co-expressed with bilin synthases and lyases [113].

Beyond light harvesting, there are many downstream photosynthetic functions that could be imprinted onto artificial components, including, for example, de novo designed RCs for biomimetic, photoinduced charge separation. Here, the light-harvested electronic excitation energy is converted into the release of electrons into an electron transport chain. Photosystem II obtains these electrons through the photolysis of water, and a prototype PSII maquette is in development by Dutton and colleagues [15]. De novo designed electron transport proteins are discussed in §4.3.

During photosynthesis photoprotection is required. In the presence of high or fluctuating light conditions, Chl and BChls can form triplet states capable of donating their energy to O_2_, producing singlet oxygen. Photosynthetic organisms have evolved various strategies to prevent singlet oxygen damage, including non-photochemical quenching (NPQ)—the dissipation of excess energy as heat, and the involvement of pigments such as carotenoids which can directly quench the singlet oxygen state and the triplet B/Chl state, followed by NPQ [114]. An artificial light-harvesting system must be able to perform similar protective mechanisms. The maquette, HP7, bound with two zinc-substituted Chl derivatives, can efficiently undergo relaxation through NPQ when the pair is photoexcited [106]. The protein environment surrounding the BChl pair can control the fate of the excitation, so the maquette can be adapted for light harvesting, leading to energy transfer, for charge separation or for energy dissipation [115]. Although this designed protein is not a single chain and therefore probably cannot be constructed *in vivo*, the design principles learned here may be adapted for a biocompatible component.

In addition to roles in photosynthesis, light-sensing proteins can perform other functions. Cryptochrome proteins contain a flavin adenine dinucleotide (FAD) cofactor that forms a radical pair (RP) when exposed to blue light. These proteins are responsible for a variety of functions, including growth towards light in plants, the control of plant development and the regulation of circadian rhythms. Cryptochromes are also thought to be involved in magnetic sensing [116]. Interconversion of the singlet and triplet states of the RP is sensed in terms of its timing and extent to achieve magnetosensitivity [117]. A flavomaquette that is capable of light-mediated magnetic field sensing has been designed and constructed [74]. This maquette was created to aid understanding of the properties required by natural proteins to sense magnetic fields. The maquette does not bear any resemblance to the natural chryptochrome fold but forms a chryptochrome-like light-active RP that is magnetically sensitive. As in natural chryptochromes, photoinduced ET from a nearby tryptophan to the flavin results in formation of the RP. The protein was expressed in and purified from *E. coli*, but the flavin cofactor, 8-bromo-riboflavin, was covalently incorporated *in vitro.* Natural cryptochromes contain a triad of tryptophans that act as a light-activated electron transport chain, which the authors aim to replicate in future designs.

### 4.3. Designed electron transport proteins

An area that has been much explored in de novo protein design is the creation of artificial ET proteins. These proteins could be integrated with respiratory processes/complexes *in vivo* to divert electrons directly towards the production of useful products or improving respiratory energy conversion in humans in the case of disease or ageing [118], or could redress redox imbalances caused as a result of metabolic engineering [119]. They may also have a role in photosynthesis; the previously discussed Mn-proteins designed by Allen and colleagues can transfer electrons to natural bacterial RCs, subsequent to illumination—which induces the RC charge-separated state. This process is analogous to the rapid reduction of RCs by natural secondary electron donors, such as cytochrome *c*_2_. Modelling results indicate that the artificial proteins bind the periplasmic face of the RC in a manner similar to cytochrome *c*_2_ [55,56].

In Nature, proteins containing iron–sulfur clusters are often involved in respiratory and photosynthetic electron transport chains. De novo ET proteins have been engineered to incorporate [4Fe-4S] clusters [120]. In a recent example, a de novo ferredoxin mimic was designed, which incorporates two [4Fe-4S] clusters in a 3-helix scaffold [121]. In a subsequent study mutants of this protein were produced to modulate redox potential and stabilize [3Fe-4S] [122]. For the construction of synthetic ET chains, or for accessing more sophisticated redox catalysis, this ability to tailor redox properties, such as potential and directionality, is an advantage. These proteins are produced by peptide synthesis and are not single chain; however, it is possible to incorporate an iron–sulfur cluster into a single-chain scaffold [73]. See also Dizicheh *et al*. [123] for a review of the incorporation of FeS clusters into both natural and artificial scaffolds.

A rate-limiting step in inter-protein ET between the multi-protein complexes of the respiratory chain is the transient encounter between the redox partners so that they are within a suitable distance for efficient ET [124]. Cytochrome *c* is a diffusible protein of the mitochondrial electron transport chain and it, along with many other natural proteins, uses complementary surface electrostatics to promote transient interactions between it and its redox partners [125]. As maquettes are highly tolerant to extensive changes in their surface residues, they provide an ideal platform for investigating the effect of differing electrostatic surface characteristics. Fry *et al.* [118] have designed a genetically encodable heme-binding maquette that can reduce cytochrome *c* at physiologically relevant (millisecond) rates. In this way, cytochrome *c* may act *in vivo* as a mediator between the artificial maquette and the natural redox partners of cytochrome *c*, who either have a net negative charge or have negatively charged surface regions. It therefore followed that the maquette with a net negative charge was demonstrated to more rapidly reduce cytochrome *c* than that with a net positive charge. This work demonstrates that, for effective biological ET, a net complementary charge is sufficient as opposed to more specific protein–protein binding interactions. In this case, it was not necessary to design a specific docking site; however, it would be advantageous to design de novo proteins that specifically interact with natural proteins.

The Pecoraro group has sought to explore whether the characteristics and properties of metals in natural proteins can be retained when placing a metal-binding site in a de novo designed protein with a different topology. Natural ET cupredoxin, CuT1, proteins contain copper-binding centres that are often found within a beta-barrel framework. The de novo protein, α3D [53,90] (see §4.1 Artificial enzymes), was modified to incorporate a copper-binding centre [91,126]. A 2HisCys(Met) metal-binding site was modelled within the 3-helix bundle, and the resulting protein can be purified from *E. coli*. The spectroscopic properties of native cupredoxins were not fully replicated in the designed protein; however, the de novo protein is capable of intramolecular ET, and can perform ET with a photosensitizer. In the future, the authors aim to optimize the structure to improve its properties, including ET efficiency, and to incorporate both an ET and a catalytic site within the same scaffold to mimic natural enzymes such as copper nitrate reductase. In recent research, the same group has used the α3D scaffold to build in a rubredoxin site, containing one iron, using a CXXC motif, with the same spectroscopic characteristics as its natural counterpart despite the site being in a different fold [127].

The ET components described above have the potential to be integrated into systems in conjunction with man-made oxidoreductases or natural proteins. This could mimic the function of natural systems, or be used to create an assortment of biomimetic components that can interact in ways not observed in Nature.

### 4.4. Artificial metal-sequestering proteins

Natural proteins may bind metal ions or compounds for purposes other than catalysis, photosynthesis and ET. In Nature, features such as teeth, bones and shells are created by biomineralization [128]; in many cases, proteins and enzymes are involved in these processes [129]. These proteins are of interest in many fields as the materials they deposit may have desirable electric, optical and magnetic properties. To this end, a de novo designed protein, Pizza, has been engineered to synthesize cadmium chloride nanocrystals. One Pizza variant, Pizza6, is a computationally designed self-assembling de novo β-propeller protein with sixfold symmetry and high thermostability, and is expressed and purified from *E. coli*. [129]. A version of Pizza was designed with histidine residues at the trimeric interfaces (nvPizza2-S16H58; see figure 1*a*), with the intention that it would only assemble in the presence of metal ions [26]. This would inevitably increase stability of the protein complex but also aid disassembly by exposure to chelators. The designed subunits consist of two propeller domains per polypeptide which spontaneously trimerize. The presence of cadmium chloride induces a dimerization of these trimers through the coordination of a 19-atom cadmium chloride nanocrystal by the symmetrically positioned histidine residues. Trimerization is observed in the absence of cadmium chloride, probably through a water molecule forming hydrogen bonds with the three histidine residues. The authors envisage introducing catalytic activity to the structure, as a water-filled tunnel is formed on one face, reminiscent of the buried tunnels containing catalytic metal ions in the active sites of catalase and superoxide dismutase.

Metalloregulatory proteins regulate ion flux and delivery within the cell while limiting levels of potentially toxic heavy metals. Pecoraro and colleagues [130] have created an artificial 3-helix bundle protein, α_3_DIV, that can bind Hg(II), Pb(II) and Cd(II). α_3_DIV is a redesigned variant of the DeGrado protein α_3_D and contains the triscysteine motif found in many metalloregulatory proteins. α_3_DIV is stable, with a more tightly packed core than α_3_D, and heavy metal binding induces further stability. The protein can be genetically encoded and expressed in *E. coli* and has been structurally characterized by nuclear magnetic resonance [92]. In a subsequent study, a fourth cysteine residue was introduced at one of two different sites in α_3_DIV to mimic the tetrathiolate binding site found in CadC, a transcriptional repressor protein that regulates the levels of intracellular Cd(II) [131]. As was identified with CadC, the tetrathiolate site in the artificial protein coordinates Cd(II) as a mixture of rapidly exchanging CdS_3_O and CdS_4_ species. Thus, the artificial metal binder acts as a model for its natural counterparts.

### 4.5. De novo designed membrane pores

Membranes provide a vital boundary between the cell and the outside world, strictly controlling what goes in and out of the cell. Natural membrane pores have many functions, including signalling and transport. Although providing selective transport across the cell membrane without compromising membrane integrity is a particular challenge, the capability of creating de novo designed membrane pores is highly advantageous, potentially facilitating the design of proteins tailored to specific ‘cargo’ molecules, through pore size and sequence. However, the design of de novo membrane protein scaffolds is hindered by the relatively small proportion of solved membrane protein structures compared with soluble proteins. Currently, de novo membrane nanopores come in many different forms, with examples constructed from proteins, DNA and organic materials [132].

A notable example of a de novo designed membrane pore is Rocker, a Zn^2+^/H^+^ antiporter, designed by the DeGrado laboratory [13,133]. Rocker is a computationally designed transporter protein which can transport Zn^2+^ or Co^2+^ ions, but not Ca^2+^, across membranes, with the concurrent antiport of protons. The phospholipid bilayer of biological membranes is impermeable to metal ions, and transporters are required to transport metal ions such as Zn^2+^ across the membrane. Rocker was designed with the aim of emulating the alternating access ‘rocker-switch’ model by which many natural transporter proteins are thought to operate, rocking between different states. It is a membrane-spanning de novo designed 4-helix protein that features two di-metal binding sites with negative cooperativity of binding. As more than 100 protons are transported per Zn^2+^ ion, Rocker's efficiency does not match that of natural proteins; however, it represents an example of how function can be achieved through a protein scaffold that is simple in comparison with its natural counterparts, with potential to engineer additional features to improve the transport efficiency. Moreover, although Rocker was produced through solid-phase peptide synthesis, production of a genetically encoded variant may be possible as the helices run antiparallel, facilitating a single-chain design. The fact that the protein can assemble in micelles and phospholipid bilayers is a promising indication that similar de novo proteins may readily insert into cell membranes.

Peptides that form pores inside membranes in acidic conditions were recently designed by the DeGrado group [134]. The computationally designed 28-residue peptides consisting of four 7-residue repeats are of sufficient length (42 Å) to span the cell membrane. The *in vivo* extracellular environment is at a pH of 7.4, and at this pH the peptides are water soluble. As the pH is lowered, the peptides bind discretely to membrane. At pH 5.5, resembling the acidic conditions found within the endosome or lysosome, the peptides assemble to form transmembrane pores. When the peptides were added to red blood cells, miRNA and ATP were selectively released, but not haemoglobin, while preserving membrane integrity. With further research and understanding, the authors envisage creating ‘selective membrane-permeating tools’ for a variety of cargo.

Another approach to selectively breach the cell membrane is to mimic the function of a virus, in which the encapsulated DNA or RNA is delivered into a host cell. Noble *et al.* [135] have created TecVir, a de novo design that forms a virus-like topology from coiled-coil peptide helices, which can transfer both RNA and DNA into human cells without cytotoxic effects. Each peptide helix was designed with one hydrophobic face and two polar interfaces, which allow it to interact with three identical neighbour helices; they pack to form a shell and can be co-assembled with DNA or RNA. Although TecVir is made *in vitro* through peptide synthesis, the assembly shows biocompatibility as it can transfect human dermal fibroblasts with a plasmid encoding eGFP without appearing to disrupt cell morphology as it is taken up by cells through endocytosis. Like the pore-forming peptides above, TecVir is pH responsive; at acidic pH, TecVir unfolds, allowing entry of the genetic cargo to the cytoplasm.

## 5. Chemical mimics of protein function

It is not exclusively artificial proteins and peptides that can mimic protein function. Some designed chemical constructs have the potential to be biocompatible, and work with or in cells. This section delineates two recently reported studies in which molecules are used for transmitting signals across membranes.

**Figure 4.**
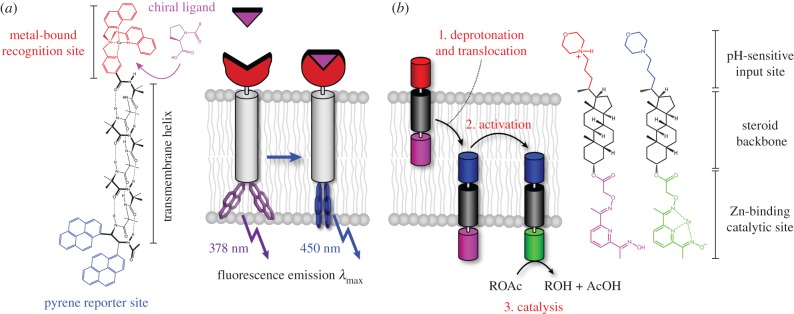
De novo transmembrane components for signalling. (*a*) A synthetic GPCR mimic [136]. The synthetic receptor consists of a ligand-binding pocket featuring a cationic metal complex (red), an Aib oligomer (grey) and a pair of pyrene molecules attached to a chiral diamine (purple and blue). This complex adopts one of two mirror image conformational states on complexation with a chiral ligand. The binding of a chiral ligand (magenta) to one end of an Aib oligomer propagates its conformational influence along the entire length. The signal is output by the conformationally responsive fluorophore (purple and blue). Thus, the binding of the cofactor perturbs the global conformation, which is reported by the fluorophore component. (*b*) A translocatable sensor [137] in which two head groups are coupled to a steroid spacer (grey). The external sensor is a protonated morpholine (red or blue), while the second head group is a neutral pyridineoxime ‘pro catalyst’ (magenta or green). When the head groups are polar (red or green), they prefer to sit in the aqueous phase; when non-polar (blue or magenta), they prefer to sit in the membrane. Binding of a zinc cofactor from within the vesicle pulls the pro-catalyst head group into the aqueous phase on the interior of the vesicle. This allows the hydrolysis of the substrate within the vesicle, generating the output signal.

Natural G-protein-coupled receptor (GPCR) signalling proteins are membrane bound. When a ligand signal is bound, a conformational change is induced in the GPCR, which initiates a signalling cascade within the cell. The Claydon group considered the minimal set of components required to create a GPCR mimic and subsequently designed a membrane-bound synthetic receptor (figure 4*a*) [136]. The receptor binding site contains a Cu(II) ion, to which a carboxylate ligand binds. This binding induces a conformational change across the receptor, consisting of a helical foldamer core derived from peptabiols, a fungal class of antibiotics. Peptabiols can insert into the membrane bilayer and contain the quaternary amino acid, α-aminoisobutyric acid (aib). Oligomers of Aib have strong helical propensity, and decamers are long enough to span the membrane bilayer. In solution these oligomers switch between two different conformational states, left- or right-handed screws, and the binding of chiral ligands determines which of these states is favoured. At the other end of the molecule is a fluorophore consisting of a pair of pyrene molecules attached to a chiral diamine. This fluorophore is sensitive to conformational change: monomeric pyrene has an emission at 378 nm, but in a certain spatial arrangement a pair of pyrene fluorophores may emit at 450 nm. Thus, the binding of the cofactor affects the conformation of the whole receptor structure, which is reported by the fluorophore component.

Another study focused on the transmission of a signal using a switch localized in either the outer or inner leaves of the membrane (figure 4*b*) [137]. Two head groups are coupled to a steroid spacer; one of the head groups, a protonated morpholine, is the external sensor and the other is a neutral pyridineoxime ‘pro-catalyst’. When the sensor head group is polar, it sits in the aqueous phase awaiting the chemical signal, while the non-polar pro-catalyst sits in the membrane. On binding a signal, the sensor head group becomes non-polar, inducing translocation across the membrane. Binding of a zinc cofactor from within the vesicle switches the pro-catalyst head group to polar, thus moving it into the aqueous vesicle interior. This activates the catalyst and allows the hydrolysis of the substrate to a fluorescent product within the vesicle. This signal transduction mechanism has been used to trigger the release of cargo from a vesicle, which may aid the development of drug delivery systems [138].

These artificial signal transducers must be assembled *in vitro*; however, as they can function in vesicle lipid bilayers, they may well be able to function *in vivo*, acting as an interface between biological and synthetic systems.

### 5.1. Making non-natural components biocompatible

While many chemical entities may have potential as biocompatible mimics of natural function, further research is required to effectively interface between biological and chemical elements to achieve full biocompatibility and functionality under physiological conditions.

A step towards achieving full biocompatibility with or within cells is to optimize the element's function under physiological conditions, and to work in partnership with natural proteins. To take an example, while natural proteins do not make use of precious metals for catalysis, these metals are used in many industrial reactions. To function as part of an artificial metalloenzyme requires strategies to aid their biocompatibility; biotin/streptavidin technology has been exploited for this purpose. The Ward group described an artificial metalloenzyme which has a biotinylated organometallic iridium catalyst anchored to an engineered streptavidin protein scaffold [139]. The streptavidin has a C-terminal artificial activating tripeptide ligand which, on proteolytic cleavage, then coordinates to the metal of the cofactor and activates the enzyme. In another study by the same group, an organometallic catalyst is integrated with natural proteins as part of an enzyme cascade to produce enantiopure amines [140]. It is difficult to fully assemble such metalloenzymes *in vivo*, in part due to the presence of inhibitors such as glutathione in cells [141]. The Ward group reported that compartmentalization within cells can be an approach to overcome this, as, for example, the periplasm contains a relative lack of glutathione. An artificial metalloenzyme for olefin metathesis (a reaction that does not occur in Nature) was expressed with a periplasmic export tag. The protein assembled *in vivo* in its functional form, and *in vivo* directed evolution was used to optimize the protein. In this case, the cofactor was biot-Ru, which is inactive until it is assembled as the mature form of the metalloenzyme [142].

## 6. Conclusion

There is still much progress to be made when it comes to fully biocompatible functional de novo proteins, although there are a few examples which fulfil these criteria [12,57,59,64]. With advances in high-throughput techniques, and accessibility of these methods, we envisage successful designs becoming more commonplace in the coming years. In addition, new methods of making synthetic components biocompatible promise to unlock *in vivo* possibilities beyond those which Nature can provide us. These biocompatible de novo units may lead to improved and tailored medical benefits through the design of drug delivery systems and therapeutic molecules. In addition, we may learn more about how natural systems work, and therefore increase our knowledge of natural design principles in order to create improved de novo components. Ultimately, we may be able to create tailor-made life forms, such as bacteria with artificial genomes that can function in environments beyond their natural niches, to create, for example, useful industrial products.

## Data accessibility

This article has no additional data.

## References

[1] Huang PS, Boyken SE, Baker D 2016 The coming of age of de novo protein design. Nature 537, 320–327. (10.1038/nature19946) 27629638

[2] Nanda V, Koder RL 2010 Designing artificial enzymes by intuition and computation. Nat. Chem. 2, 15–24. (10.1038/nchem.473) 21124375 PMC3443871

[3] Reetz MT 2013 Biocatalysis in organic chemistry and biotechnology: past, present, and future. J. Am. Chem. Soc. 135, 12 480–12 496. (10.1021/ja405051f) 23930719

[4] Sheldon RA, Woodley JM 2018 Role of biocatalysis in sustainable chemistry. Chem. Rev. 118, 801–838. (10.1021/acs.chemrev.7b00203) 28876904

[5] Siegel JB et al. 2010 Computational design of an enzyme catalyst for a stereoselective bimolecular Diels-Alder reaction. Science 329, 309–313. (10.1126/science.1190239) 20647463 PMC3241958

[6] Channon K, Bromley EHC, Woolfson DN 2008 Synthetic biology through biomolecular design and engineering. Curr. Opin Struct. Biol. 18, 491–498. (10.1016/j.sbi.2008.06.006) 18644449

[7] Armstrong CT, Watkins DW, Anderson JL.R 2013 Constructing manmade enzymes for oxygen activation. Dalton Trans. 42, 3136–3150. (10.1039/c2dt32010j) 23076271

[8] Muller HJ 1964 The relation of recombination to mutational advance. Mutat. Res. 1, 2–9. (10.1016/0027-5107(64)90047-8) 14195748

[9] Dutton PL, Moser CC 2011 Engineering enzymes. Faraday Discuss. 148, 443–448. (10.1039/c005523a) 21322497 PMC4073794

[10] Koder RL, Dutton PL 2006 Intelligent design: the de novo engineering of proteins with specified functions. Dalton Trans. 2006, 3045–3051. (10.1039/b514972j) 16786062

[11] Lichtenstein BR et al. 2012 Engineering oxidoreductases: maquette proteins designed from scratch. Biochem. Soc. Trans. 40, 561–566. (10.1042/bst20120067) 22616867 PMC3525474

[12] Watkins DW et al. 2017 Construction and *in vivo* assembly of a catalytically proficient and hyperthermostable de novo enzyme. Nat. Commun. 8, 358 (10.1038/s41467-017-00541-4) 28842561 PMC5572459

[13] Joh NH, Wang T, Bhate MP, Acharya R, Wu YB, Grabe M, Hong M, Grigoryan G, DeGrado WF 2014 De novo design of a transmembrane Zn^2+^-transporting four-helix bundle. Science 346, 1520–1524. (10.1126/science.1261172) 25525248 PMC4400864

[14] Watkins DW, Armstrong CT, Anderson JLR 2014 De novo protein components for oxidoreductase assembly and biological integration. Curr. Opin Chem. Biol. 19, 90–98. (10.1016/j.cbpa.2014.01.016) 24607598

[15] Ennist N, Mancini JB, Auman D, Bialas CJ, Iwanicki M, Esipova T, Discher BC, Moser C, Dutton PL 2017 Maquette strategy for creation of light- and redox-active proteins. In Photosynthesis and bioenergetics (eds Barber J, Ruban AV), pp. 1–33. Singapore: World Scientific.

[16] Regan L, Degrado WF 1988 Characterization of a helical protein designed from 1st principles. Science 241, 976–978. (10.1126/science.3043666) 3043666

[17] Cohen C, Parry DA.D 1990 Alpha-helical coiled coils and bundles—how to design an alpha-helical protein. Proteins 7, 1–15. (10.1002/prot.340070102) 2184436

[18] Crick FHC 1953 The packing of alpha-helices—simple coiled-coils. Acta Crystallogr. 6, 689–697. (10.1107/s0365110×53001964)

[19] Woolfson DN 2005 The design of coiled-coil structures and assemblies. Adv. Protein Chem. 70, 79 (10.1016/s0065-3233(04)70004-2) 15837514

[20] Woolfson DN, Bartlett GJ, Bruning M, Thomson AR 2012 New currency for old rope: from coiled-coil assemblies to alpha-helical barrels. Curr. Opin Struct. Biol. 22, 432–441. (10.1016/j.sbi.2012.03.002) 22445228

[21] Kim PS 1988 Passing the 1st milestone in protein design. Protein Eng. 2, 249–250. (10.1093/protein/2.4.249) 3249742

[22] Kamtekar S, Schiffer JM, Xiong HY, Babik JM, Hecht MH 1993 Protein design by binary patterning of polar and nonpolar amino-acids. Science 262, 1680–1685. (10.1126/science.8259512) 8259512

[23] Farid TA et al. 2013 Elementary tetrahelical protein design for diverse oxidoreductase functions. Nat. Chem. Biol. 9, 826 (10.1038/nchembio.1362) 24121554 PMC4034760

[24] Anderson JLR et al. 2014 Constructing a man-made c-type cytochrome maquette *in vivo*: electron transfer, oxygen transport and conversion to a photoactive light harvesting maquette. Chem. Sci. 5, 507–514. (10.1039/c3sc52019f) 24634717 PMC3952003

[25] Boyken SE et al. 2016 De novo design of protein homo-oligomers with modular hydrogen bond network-mediated specificity. Protein Sci. 25, 52–53. 10.1126/science.aad8865 PMC5497568 27151862

[26] Voet ARD, Noguchi H, Addy C, Zhang KYJ, Tame JRH 2015 Biomineralization of a cadmium chloride nanocrystal by a designed symmetrical protein. Angew. Chem. Int. Ed. 54, 9857–9860. (10.1002/anie.201503575) 26136355

[27] Ennist NM, Zhao Z, Stayrook SE, Dutton PL, Moser CC. In preparation. Design, structure, and action of an artificial photosynthetic reaction center protein.

[28] Burton AJ, Thomson AR, Dawson WM, Brady RL, Woolfson DN 2016 Installing hydrolytic activity into a completely de novo protein framework. Nat. Chem. 8, 837–844. (10.1038/nchem.2555) 27554410

[29] Ulas G, Lemmin T, Wu YB, Gassner GT, DeGrado WF 2016 Designed metalloprotein stabilizes a semiquinone radical. Nat. Chem. 8, 354–359. (10.1038/nchem.2453) 27001731 PMC4857601

[30] MacDonald JT, Kabasakal BV, Godding D, Kraatz S, Henderson L, Barber J, Freemont PS, Murray JW 2016 Synthetic beta-solenoid proteins with the fragment-free computational design of a beta-hairpin extension. Proc. Natl Acad. Sci. USA 113, 10 346–10 351. (10.1073/pnas.1525308113) 27573845 PMC5027453

[31] Huang PS, Feldmeier K, Parmeggiani F, Velasco DAF, Hocker B, Baker D 2016 De novo design of a four-fold symmetric TIM-barrel protein with atomic-level accuracy. Nat. Chem. Biol. 12, 29 (10.1038/nchembio.1966) 26595462 PMC4684731

[32] Parmeggiani F, Huang PS 2017 Designing repeat proteins: a modular approach to protein design. Curr. Opin Struct. Biol. 45, 116–123. (10.1016/j.sbi.2017.02.001) 28267654

[33] MacDonald JT, Maksimiak K, Sadowski MI, Taylor WR 2010 De novo backbone scaffolds for protein design. Proteins 78, 1311–1325. (10.1002/prot.22651) 20017215 PMC2841848

[34] MacDonald JT, Kelley LA, Freemont PS 2013 Validating a coarse-grained potential energy function through protein loop modelling. PLoS ONE 8, e0065770 (10.1371/journal.pone.0065770) PMC3688807 23824634

[35] Moore BL, Kelley LA, Barber J, Murray JW, MacDonald JT 2013 High-quality protein backbone reconstruction from alpha carbons using Gaussian mixture models. J. Comput. Chem. 34, 1881–1889. (10.1002/jcc.23330) 23703289

[36] Wierenga RK 2001 The TIM-barrel fold: a versatile framework for efficient enzymes. FEBS Lett. 492, 193–198. 11257493 10.1016/s0014-5793(01)02236-0

[37] Hocker B, Jurgens C, Wilmanns M, Sterner R 2001 Stability, catalytic versatility and evolution of the (beta alpha)(8)-barrel fold. Curr. Opin Biotechnol. 12, 376–381. (10.1016/s0958-1669(00)00230-5) 11551466

[38] Nagano N, Orengo CA, Thornton JM 2002 One fold with many functions: the evolutionary relationships between TIM barrel families based on their sequences, structures and functions. J. Mol. Biol. 321, 741–765. (10.1016/s0022-2836(02)00649-6) 12206759

[39] Whitley P, Nilsson I, von Heijne G 1994 De novo design of integral membrane proteins. Nat. Struct. Biol. 1, 858–862. 7773774 10.1038/nsb1294-858

[40] Goparaju G, Fry BA, Chobot SE, Wiedman G, Moser CC, Dutton PL, Discher BM 2016 First principles design of a core bioenergetic transmembrane electron-transfer protein. Biochim. Biophys. Acta 1857, 503–512. (10.1016/j.bbabio.2015.12.002) 26672896 PMC4846532

[41] Moser CC, Sheehan MM, Ennist NM, Kodali G, Bialas C, Englander MT, Discher BM, Dutton PL 2016 De novo construction of redox active proteins. Methods Enzymol. 580, 365–388. (10.1016/bs.mie.2016.05.048) 27586341 PMC5123760

[42] Lu P et al. 2018 Accurate computational design of multipass transmembrane proteins. Science 359, 1042–1046. (10.1126/science.aaq1739) 29496880 PMC7328376

[43] Zozulia O, Dolan MA, Korendovych IV 2018 Catalytic peptide assemblies. Chem. Soc. Rev. 47, 3621–3639. (10.1039/c8cs00080h) 29594277 PMC6027653

[44] Akcapinar GB, Sezerman OU 2017 Computational approaches for de novo design and redesign of metal-binding sites on proteins. Biosci. Rep. 37, pii BSR20160179 (10.1042/BSR20160179) PMC5482196 28167677

[45] Negron C, Fufezan C, Koder RL 2009 Geometric constraints for porphyrin binding in helical protein binding sites. Proteins 74, 400–416. (10.1002/prot.22143) 18636480 PMC2844854

[46] Braun P, Goldberg E, Negron C, von Jan M, Xu F, Nanda V, Koder RL, Noy D 2011 Design principles for chlorophyll-binding sites in helical proteins. Proteins 79, 463–476. (10.1002/prot.22895) 21117078 PMC6298425

[47] Hecht MH, Das A, Go A, Bradley LH, Wei YN 2004 De novo proteins from designed combinatorial libraries. Protein Sci. 13, 1711–1723. (10.1110/ps.04690804) 15215517 PMC2279937

[48] Fisher MA, McKinley KL, Bradley LH, Viola SR, Hecht MH 2011 De novo designed proteins from a library of artificial sequences function in Escherichia coli and enable cell growth. PLoS ONE 6, e15364 (10.1371/journal.pone.0015364) 21245923 PMC3014984

[49] Digianantonio KM, Hecht MH 2016 A protein constructed de novo enables cell growth by altering gene regulation. Proc. Natl Acad. Sci. USA 113, 2400–2405. (10.1073/pnas.1600566113) 26884172 PMC4780649

[50] Hoegler KJ, Hecht MH 2016 A de novo protein confers copper resistance in Escherichia coli. Protein Sci. 25, 1249–1259. (10.1002/pro.2871) 26748884 PMC4918413

[51] Digianantonio KM, Korolev M, Hecht MH 2017 A non-natural protein rescues cells deleted for a key enzyme in central metabolism. ACS Synth. Biol. 6, 694–700. (10.1021/acssynbio.6b00336) 28055179

[52] Smith BA, Mularz AE, Hecht MH 2015 Divergent evolution of a bifunctional de novo protein. Protein Sci. 24, 246–252. (10.1002/pro.2611) 25420677 PMC4315662

[53] Walsh STR, Cheng H, Bryson JW, Roder H, DeGrado WF 1999 Solution structure and dynamics of a de novo designed three-helix bundle protein. Proc. Natl Acad. Sci. USA 96, 5486–5491. (10.1073/pnas.96.10.5486) 10318910 PMC21886

[54] Cangelosi VM, Deb A, Penner-Hahn JE, Pecoraro VL 2014 A de novo designed metalloenzyme for the hydration of CO_2_. Angew. Chem. Int. Ed. 53, 7900–7903. (10.1002/anie.201404925) PMC4107010 24943466

[55] Olson TL, Espiritu E, Edwardraja S, Simmons CR, Williams JC, Ghirlanda G, Allen JP 2016 Design of dinuclear manganese cofactors for bacterial reaction centers. Biochim. Biophys. Acta 1857, 539–547. (10.1016/j.bbabio.2015.09.003) 26392146

[56] Olson TL, Espiritu E, Edwardraja S, Canarie E, Flores M, Williams JC, Ghirlanda G, Allen JP 2017 Biochemical and spectroscopic characterization of dinuclear Mn-sites in artificial four-helix bundle proteins. Biochim. Biophys. Acta 1858, 945–954. (10.1016/j.bbabio.2017.08.013) 28882760

[57] Donnelly AE, Murphy GS, Digianantonio KM, Hecht MH 2018 A de novo enzyme catalyzes a life-sustaining reaction in Escherichia coli. Nat. Chem. Biol. 14, 253–255. (10.1038/nchembio.2550) 29334382

[58] Strauch EM et al. 2017 Computational design of trimeric influenza-neutralizing proteins targeting the hemagglutinin receptor binding site. Nat. Biotechnol. 35, 667–671. (10.1038/nbt.3907) 28604661 PMC5512607

[59] Chevalier A et al. 2017 Massively parallel de novo protein design for targeted therapeutics. Nature 550, 74 (10.1038/nature23912) 28953867 PMC5802399

[60] Berger S et al. 2016 Computationally designed high specificity inhibitors delineate the roles of BCL2 family proteins in cancer. Elife 5, e20352 (10.7554/elife.20352) 27805565 PMC5127641

[61] Procko E et al. 2014 A Computationally designed inhibitor of an Epstein-Barr viral Bcl-2 protein induces apoptosis in infected cells. Cell 157, 1644–1656. (10.1016/j.cell.2014.04.034) 24949974 PMC4079535

[62] Bhardwaj G et al. 2016 Accurate de novo design of hyperstable constrained peptides. Nature 538, 329 (10.1038/nature19791) 27626386 PMC5161715

[63] Heddle JG, Chakraborti S, Iwasaki K 2017 Natural and artificial protein cages: design, structure and therapeutic applications. Curr. Opin Struct. Biol. 43, 148–155. (10.1016/j.sbi.2017.03.007) 28359961

[64] Butterfield GL et al. 2017 Evolution of a designed protein assembly encapsulating its own RNA genome. Nature 552, 415 (10.1038/nature25157) 29236688 PMC5927965

[65] Sutherland GA et al. 2018 Probing the quality control mechanism of the Escherichia coli twin-arginine translocase with folding variants of a de novo-designed heme protein. J. Biol. Chem. 293, 6672–6681. (10.1074/jbc.RA117.000880) 29559557 PMC5936819

[66] Matsuzaki R, Fukui T, Sato H, Ozaki Y, Tanizawa K 1994 Generation of the topa quinone cofactor in bacterial monoamine-oxidase by cupric ion-dependent autooxidation of a specific tyrosyl residue. FEBS Lett. 351, 360–364. (10.1016/0014-5793(94)00884-1) 8082796

[67] Robertson DE et al. 1994 Design and synthesis of multi-heme proteins. Nature 368, 425–431. (10.1038/368425a0) 8133888

[68] Watkins DW, Armstrong CT, Beesley JL, Marsh JE, Jenkins JMX, Sessions RB, Mann S, Anderson JLR 2016 A suite of de novo c-type cytochromes for functional oxidoreductase engineering. Biochim. Biophys. Acta 1857, 493–502. (10.1016/j.bbabio.2015.11.003) 26556173

[69] Poulos TL 2014 Heme enzyme structure and function. Chem. Rev. 114, 3919–3962. (10.1021/cr400415k) 24400737 PMC3981943

[70] Das A, Hecht MH 2007 Peroxidase activity of de novo heme proteins immobilized on electrodes. J. Inorg. Biochem. 101, 1820–1826. (10.1016/j.jinorgbio.2007.07.024) 17765314 PMC2080791

[71] Moffet DA, Certain LK, Smith AJ, Kessel AJ, Beckwith KA, Hecht MH 2000 Peroxidase activity in heme proteins derived from a designed combinatorial library. J. Am. Chem. Soc. 122, 7612–7613. (10.1021/ja001198q)

[72] Woodward JJ, Martin NI, Marletta MA 2007 An Escherichia coli expression-based method for heme substitution. Nat. Methods 4, 43–45. (10.1038/nmeth984) 17187078

[73] Grzyb J, Xu F, Weiner L, Reijerse EJ, Lubitz W, Nanda V, Noy D 2010 De novo design of a non-natural fold for an iron-sulfur protein: alpha-helical coiled-coil with a four-iron four-sulfur cluster binding site in its central core. Biochim. Biophys. Acta 1797, 406–413. (10.1016/j.bbabio.2009.12.012) 20035711

[74] Bialas C et al. 2016 Engineering an artificial flavoprotein magnetosensor. J. Am. Chem. Soc. 138, 16 584–16 587. (10.1021/jacs.6b09682) PMC5711731 27958724

[75] Hammes-Schiffer S 2013 Catalytic efficiency of enzymes: a theoretical analysis. Biochemistry 52, 2012–2020. (10.1021/bi301515j) 23240765 PMC3619019

[76] Alberty RA, Hammes GG 1958 Application of the theory of diffusion-controlled reactions to enzyme kinetics. J. Phys. Chem. 62, 154–159. (10.1021/j150560a005)

[77] Kamerlin SCL, Warshel A 2010 At the dawn of the 21st century: is dynamics the missing link for understanding enzyme catalysis? Proteins 78, 1339–1375. (10.1002/prot.22654) 20099310 PMC2841229

[78] Benkovic SJ, Hammes-Schiffer S 2003 A perspective on enzyme catalysis. Science 301, 1196–1202. (10.1126/science.1085515) 12947189

[79] Fersht A 2017 Structure and mechanism in protein science: a guide to enzyme catalysis and protein folding. Singapore: World Scientific.

[80] Hempel J, Kuo I, Perozich J, Wang BC, Lindahl R, Nicholas H 2001 Aldehyde dehydrogenase—maintaining critical active site geometry at motif 8 in the class 3 enzyme. Eur. J. Biochem. 268, 722–726. (10.1046/j.1432-1327.2001.01926.x) 11168411

[81] Kim TW, Brieba LG, Ellenberger T, Kool ET 2006 Functional evidence for a small and rigid active site in a high fidelity DNA polymerase—probing T7 DNA polymerase with variably sized base pairs. J. Biol. Chem. 281, 2289–2295. (10.1074/jbc.M510744200) 16311403

[82] Grayson KJ, Anderson JR 2018 The ascent of man(made oxidoreductases). Curr. Opin Struct. Biol. 51, 149–155. (10.1016/j.sbi.2018.04.008) 29754103 PMC6227378

[83] Lombardi A, Summa CM, Geremia S, Randaccio L, Pavone V, DeGrado WF 2000 Retrostructural analysis of metalloproteins: application to the design of a minimal model for diiron proteins. Proc. Natl Acad. Sci. USA 97, 6298–6305. (10.1073/pnas.97.12.6298) 10841536 PMC18597

[84] Feig AL, Lippard SJ 1994 Reactions of nonheme iron(ii) centers with dioxygen in biology and chemistry. Chem. Rev. 94, 759–805. (10.1021/cr00027a011)

[85] Chino M, Maglio O, Nastri F, Pavone V, DeGrado WF, Lombardi A 2015 Artificial diiron enzymes with a de novo designed four-helix bundle structure. Eur. J. Inorg. Chem. 2015, 3371–3390. (10.1002/ejic.201500470) 27630532 PMC5019575

[86] Reig AJ et al. 2012 Alteration of the oxygen-dependent reactivity of de novo Due Ferri proteins. Nat. Chem. 4, 900–906. (10.1038/nchem.1454) 23089864 PMC3568993

[87] Snyder RA, Butch SE, Reig AJ, DeGrado WF, Solomon EI 2015 Molecular-level insight into the differential oxidase and oxygenase reactivities of de novo Due Ferri proteins. J. Am. Chem. Soc. 137, 9302–9314. (10.1021/jacs.5b03524) 26090726 PMC4843592

[88] Snyder RA, Betzu J, Butch SE, Reig AJ, DeGrado WF, Solomon EI 2015 Systematic perturbations of binuclear non-heme iron sites: structure and dioxygen reactivity of de novo Due Ferri proteins. Biochemistry 54, 4637–4651. (10.1021/acs.biochem.5b00324) 26154739 PMC4857603

[89] Zastrow ML, Peacock AFA, Stuckey JA, Pecoraro VL 2012 Hydrolytic catalysis and structural stabilization in a designed metalloprotein. Nat. Chem. 4, 118–123. (10.1038/nchem.1201) PMC3270697 22270627

[90] Bryson JW, Desjarlais JR, Handel TM, DeGrado WF 1998 From coiled coils to small globular proteins: design of a native-like three-helix bundle. Protein Sci. 7, 1404–1414. (10.1002/pro.5560070617) 9655345 PMC2144029

[91] Plegaria JS, Duca M, Tard C, Friedlander TJ, Deb A, Penner-Hahn JE, Pecoraro VL 2015 De novo design and characterization of copper metallopeptides inspired by native cupredoxins. Inorg. Chem. 54, 9470–9482. (10.1021/acs.inorgchem.5b01330) 26381361 PMC5241702

[92] Plegaria JS, Dzul SP, Zuiderweg ERP, Stemmler TL, Pecoraro VL 2015 Apoprotein structure and metal binding characterization of a de novo designed peptide, alpha 3DIV, that sequesters toxic heavy metals. Biochemistry 54, 2858–2873. (10.1021/acs.biochem.5b00064) 25790102 PMC4492461

[93] Der BS, Edwards DR, Kuhlman B 2012 Catalysis by a de novo zinc-mediated protein interface: implications for natural enzyme evolution and rational enzyme engineering. Biochemistry 51, 3933–3940. (10.1021/bi201881p) 22510088 PMC3348550

[94] Song WJ, Tezcan FA 2014 A designed supramolecular protein assembly with *in vivo* enzymatic activity. Science 346, 1525–1528. (10.1126/science.1259680) 25525249

[95] Westerlund K, Berry BW, Privett HK, Tommos C 2005 Exploring amino-acid radical chemistry: protein engineering and de novo design. Biochim. Biophys. Acta 1707, 103–116. (10.1016/j.bbabio.2004.02.013) 15721609

[96] Ravichandran KR, Liang L, Stubbe J, Tommos C 2013 Formal reduction potential of 3,5-difluorotyrosine in a structured protein: insight into multistep radical transfer. Biochemistry 52, 8907–8915. (10.1021/bi401494f) 24228716 PMC4076196

[97] Tommos C, Valentine KG, Martinez-Rivera MC, Liang L, Moorman VR 2013 Reversible phenol oxidation and reduction in the structurally well-defined 2-mercaptophenol-alpha C-3 protein. Biochemistry 52, 1409–1418. (10.1021/bi301613p) 23373469 PMC3848601

[98] Marcos E et al. 2017 Principles for designing proteins with cavities formed by curved beta sheets. Science 355, 201–206. (10.1126/science.aah7389) 28082595 PMC5588894

[99] Polizzi NF, Wu YB, Lemmin T, Maxwell AM, Zhang SQ, Rawson J, Beratan DN, Therien MJ, DeGrado WF 2017 De novo design of a hyperstable non-natural protein-ligand complex with sub-angstrom accuracy. Nat. Chem. 9, 1157–1164. (10.1038/nchem.2846) 29168496 PMC5859929

[100] Noy D, Discher BM, Rubtsov IV, Hochstrasser RA, Dutton PL 2005 Design of amphiphilic protein maquettes: enhancing maquette functionality through binding of extremely hydrophobic cofactors to lipophilic domains. Biochemistry 44, 12 344–12 354. (10.1021/bi050696e) PMC2597482 16156647

[101] Discher BM, Noy D, Strzalka J, Ye SX, Moser CC, Lear JD, Blasie JK, Dutton PL 2005 Design of amphiphilic protein maquettes: controlling assembly, membrane insertion, and cofactor interactions. Biochemistry 44, 12 329–12 343. (10.1021/bi050695m) PMC2574520 16156646

[102] Korendovych IV, Senes A, Kim YH, Lear JD, Fry HC, Therien MJ, Blasie JK, Walker FA, DeGrado WF 2010 De novo design and molecular assembly of a transmembrane diporphyrin-binding protein complex. J. Am. Chem. Soc. 132, 15 516–15 518. (10.1021/ja107487b) PMC3016712 20945900

[103] Vonheijne G 1986 The distribution of positively charged residues in bacterial inner membrane-proteins correlates with the trans-membrane topology. EMBO J. 5, 3021–3027. 16453726 10.1002/j.1460-2075.1986.tb04601.x PMC1167256

[104] Blankenship RE et al. 2011 Comparing photosynthetic and photovoltaic efficiencies and recognizing the potential for improvement. Science 332, 805–809. (10.1126/science.1200165) 21566184

[105] Grayson KJ et al. 2017 Augmenting light coverage for photosynthesis through YFP-enhanced charge separation at the Rhodobacter sphaeroides reaction centre. Nat. Commun. 8, 13 972 (10.1038/ncomms13972) 28054547 PMC5512671

[106] Cohen-Ofri I, van Gastel M, Grzyb J, Brandis A, Pinkas I, Lubitz W, Noy D 2011 Zinc-bacteriochlorophyllide dimers in de novo designed four-helix bundle proteins. A model system for natural light energy harvesting and dissipation. J. Am. Chem. Soc. 133, 9526–9535. (10.1021/ja202054m) 21563814

[107] Koder RL, Valentine KG, Cerda J, Noy D, Smith KM, Wand AJ, Dutton PL 2006 Nativelike structure in designed four alpha-helix bundles driven by buried polar interactions. J. Am. Chem. Soc. 128, 14 450–14 451. (10.1021/ja064883r) 17090015

[108] Agostini A, Palm DM, Schmitt FJ, Albertini M, Valentin MD, Paulsen H, Carbonera D 2017 An unusual role for the phytyl chains in the photoprotection of the chlorophylls bound to water-soluble chlorophyll-binding proteins. Sci. Rep. 7, 7504 (10.1038/s41598-017-07874-6) 28790428 PMC5548782

[109] Bednarczyk D, Takahashi S, Satoh H, Noy D 2015 Assembly of water-soluble chlorophyll-binding proteins with native hydrophobic chlorophylls in water-in-oil emulsions. Biochim. Biophys. Acta 1847, 307–313. (10.1016/j.bbabio.2014.12.003) 25511505

[110] Rau HK, Snigula H, Struck A, Robert B, Scheer H, Haehnel W 2001 Design, synthesis and properties of synthetic chlorophyll proteins. Eur. J. Biochem. 268, 3284–3295. (10.1046/j.1432-1327.2001.02231.x) 11389731

[111] Kodali G et al. 2017 Design and engineering of water-soluble light-harvesting protein maquettes. Chem. Sci. 8, 316–324. (10.1039/c6sc02417c) 28261441 PMC5330312

[112] Zeng XL, Tang K, Zhou N, Zhou M, Hou HJM, Scheer H, Zhao KH, Noy D 2013 Bimodal intramolecular excitation energy transfer in a multichromophore photosynthetic model system: hybrid fusion proteins comprising natural phycobilin- and artificial chlorophyll-binding domains. J. Am. Chem. Soc. 135, 13 479–13 487. (10.1021/ja405617c) 23941594

[113] Mancini JA, Kodali G, Jiang JB, Reddy KR, Lindsey JS, Bryant DA, Dutton PL, Moser CC 2017 Multi-step excitation energy transfer engineered in genetic fusions of natural and synthetic light-harvesting proteins. J. R. Soc. Interface 14, 20160896 (10.1098/rsif.2016.0896) 28179548 PMC5332574

[114] Cogdell RJ, Frank HA 1987 How carotenoids function in photosynthetic bacteria. Biochim. Biophys. Acta 895, 63–79. 3332774 10.1016/s0304-4173(87)80008-3

[115] Wahadoszamen M, Margalit I, Ara AM, van Grondelle R, Noy D 2014 The role of charge-transfer states in energy transfer and dissipation within natural and artificial bacteriochlorophyll proteins. Nat. Commun. 5, 5287 (10.1038/ncomms6287) 25342121 PMC4255223

[116] Chaves I et al. 2011 The cryptochromes: blue light photoreceptors in plants and animals. Annu. Rev. Plant Biol. 62, 335–364. (10.1146/annurev-arplant-042110-103759) 21526969

[117] Rodgers CT, Hore PJ 2009 Chemical magnetoreception in birds: the radical pair mechanism. Proc. Natl Acad. Sci. USA 106, 353–360. (10.1073/pnas.0711968106) 19129499 PMC2626707

[118] Fry BA, Solomon LA, Dutton PL, Moser CC 2016 Design and engineering of a man-made diffusive electron-transport protein. Biochim. Biophys. Acta 1857, 513–521. (10.1016/j.bbabio.2015.09.008) 26423266 PMC4910091

[119] Thakker C, Martinez I, Li W, San KY, Bennett GN 2015 Metabolic engineering of carbon and redox flow in the production of small organic acids. J. Ind. Microbiol. Biotechnol. 42, 403–422. (10.1007/s10295-014-1560-y) 25502283

[120] Nanda V, Senn S, Pike DH, Rodriguez-Granillo A, Hansen WA, Khare SD, Noy D 2016 Structural principles for computational and de novo design of 4Fe-4S metalloproteins. Biochim. Biophys. Acta 1857, 531–538. (10.1016/j.bbabio.2015.10.001) 26449207 PMC5389887

[121] Roy A, Sommer DJ, Schmitz RA, Brown CL, Gust D, Astashkin A, Ghirlanda G 2014 A de novo designed 2 4Fe-4S ferredoxin mimic mediates electron transfer. J. Am. Chem. Soc. 136, 17 343–17 349. (10.1021/ja510621e) 25437708

[122] Sommer DJ, Roy A, Astashkin A, Ghirlanda G 2015 Modulation of cluster incorporation specificity in a de novo iron-sulfur cluster binding peptide. Biopolymers 104, 412–418. (10.1002/bip.22635) 25808361

[123] Dizicheh ZB, Halloran N, Asma W, Ghirlanda G 2017 De novo design of iron-sulfur proteins. Methods Enzymol. 595, 33–53. (10.1016/bs.mie.2017.07.014) 28882205

[124] McLendon G, Hake R 1992 Interprotein electron-transfer. Chem. Rev. 92, 481–490. (10.1021/cr00011a007)

[125] Volkov AN, van Nuland NAJ 2012 Electron transfer interactome of cytochrome c. PLoS Comput. Biol. 8, e1002807 (10.1371/journal.pcbi.1002807) 23236271 PMC3516563

[126] Plegaria JS, Herrero C, Quaranta A, Pecoraro VL 2016 Electron transfer activity of a de novo designed copper center in a three-helix bundle fold. Biochim. Biophys. Acta 1857, 522–530. (10.1016/j.bbabio.2015.09.007) 26427552 PMC5233711

[127] Tebo AG et al. 2018 Development of a rubredoxin-type center embedded in a de dovo-designed three-helix bundle. Biochemistry 57, 2308–2316. (10.1021/acs.biochem.8b00091) 29561598 PMC5982097

[128] Weiner S, Dove PM 2003 An overview of biomineralization processes and the problem of the vital effect. Biomineralization 54, 1–29. (10.2113/0540001)

[129] Voet ARD, Noguchi H, Addy C, Simoncini D, Terada D, Unzai S, Park SY, Zhang KYJ, Tame JRH 2014 Computational design of a self-assembling symmetrical beta-propeller protein. Proc. Natl Acad. Sci. USA 111, 15 102–15 107. (10.1073/pnas.1412768111) 25288768 PMC4210308

[130] Chakraborty S, Kravitz JY, Thulstrup PW, Hemmingsen L, DeGrado WF, Pecoraro VL 2011 Design of a three-helix bundle capable of binding heavy metals in a triscysteine environment. Angew. Chem. Int. Ed. 50, 2049–2053. (10.1002/anie.201006413) PMC3058785 21344549

[131] Tebo AG, Hemmingsen L, Pecoraro VL 2015 Variable primary coordination environments of Cd(II) binding to three helix bundles provide a pathway for rapid metal exchange. Metallomics 7, 1555–1561. (10.1039/c5mt00228a) 26503746 PMC5250511

[132] Howorka S 2017 Building membrane nanopores. Nat. Nanotechnol. 12, 619–630. (10.1038/nnano.2017.99) 28681859

[133] Joh NH, Grigoryan G, Wu YB, DeGrado WF 2017 Design of self-assembling transmembrane helical bundles to elucidate principles required for membrane protein folding and ion transport. Phil. Trans. R. Soc. B 372, 20160214 (10.1098/rstb.2016.0214) 28630154 PMC5483517

[134] Zhang Y et al. 2015 Computational design and experimental characterization of peptides intended for pH-dependent membrane insertion and pore formation. ACS Chem. Biol. 10, 1082–1093. (10.1021/cb500759p) 25630033 PMC4843813

[135] Noble JE, De Santis E, Ravi J, Lamarre B, Castelletto V, Mantell J, Ray S, Ryadnov MG 2016 A de novo virus-like topology for synthetic virions. J. Am. Chem. Soc. 138, 12 202–12 210. (10.1021/jacs.6b05751) 27585246

[136] Lister FGA, Le Bailly BAF, Webb SJ, Clayden J 2017 Ligand-modulated conformational switching in a fully synthetic membrane-bound receptor. Nat. Chem. 9, 420–425. (10.1038/nchem.2736)

[137] Langton MJ, Keymeulen F, Ciaccia M, Williams NH, Hunter CA 2017 Controlled membrane translocation provides a mechanism for signal transduction and amplification. Nat. Chem. 9, 426–430. (10.1038/nchem.2678) 28430205

[138] Langton MJ, Scriven LM, Williams NH, Hunter CA 2017 Triggered release from lipid bilayer vesicles by an artificial transmembrane signal transduction system. J. Am. Chem. Soc. 139, 15 768–15 773. (10.1021/jacs.7b07747) 28876061

[139] Liu Z, Lebrun V, Kitanosono T, Mallin H, Kohler V, Haussinger D, Hilvert D, Kobayashi S, Ward TR 2016 Upregulation of an artificial zymogen by proteolysis. Angew. Chem. Int. Ed. 55, 11 587–11 590. (10.1002/anie.201605010) 27529471

[140] Okamoto Y, Kohler V, Ward TR 2016 An NAD(P)H-dependent artificial transfer hydrogenase for multienzymatic cascades. J. Am. Chem. Soc. 138, 5781–5784. (10.1021/jacs.6b02470) 27100673

[141] Wilson YM, Durrenberger M, Nogueira ES, Ward TR 2014 Neutralizing the detrimental effect of glutathione on precious metal catalysts. J. Am. Chem. Soc. 136, 8928–8932. (10.1021/ja500613n) 24918731

[142] Jeschek M, Reuter R, Heinisch T, Trindler C, Klehr J, Panke S, Ward TR 2016 Directed evolution of artificial metalloenzymes for *in vivo* metathesis. Nature 537, 661 (10.1038/nature19114) 27571282

